# Predicting carotid plaques in metabolic dysfunction-associated steatotic liver disease using machine learning and SHAP interpretation

**DOI:** 10.1038/s41598-025-19959-8

**Published:** 2025-10-15

**Authors:** Shu-Mei Zhai, Xiao-Long Wang, Han Zhang, Yu-Qiang Zuo

**Affiliations:** 1 Department of Ultrasound, The 2nd Hospital of Hebei Medical University, Shijiazhuang, Hebei Province China; 2 Department of Information Center, The 2nd Hospital of Hebei Medical University, Shijiazhuang, Hebei Province China; 3 Department of Physical Examination Center, The 2nd Hospital of Hebei Medical University, Shijiazhuang, Hebei Province China

**Keywords:** Carotid plaque, Metabolic dysfunction-associated steatotic liver disease, Machine learning, Shapley additive explanations, Diseases, Health care, Medical research

## Abstract

Cardiovascular disease (CVD) remains the most common cause of death worldwide. Carotid plaque is an indicator of subclinical CVDs. Metabolic dysfunction-associated steatotic liver disease (MASLD) is a risk factor for atherosclerotic CVDs. We aimed to develop and validate a predictive model for carotid plaque occurrence in annual health check-up populations, to integrate health check-up indicators with machine learning (ML) algorithms and LASSO-based feature selection and leverage advanced interpretability frameworks to elucidate the contribution of individual risk factors. In this retrospective cohort study, we enrolled 4,973 MASLD patients, among whom 1,178 were diagnosed with carotid plaques using carotid ultrasound. Collected baseline data included ​demographic indicators, ​clinical histories, blood ​biochemical parameters, and liver function test indicators. A predictive model for carotid plaques was developed and validated using five ML algorithms. Model performance was evaluated based on the​ area under the curve, ​sensitivity, ​specificity, ​accuracy, and ​F1 Score. For model interpretability, we adopted the ​Shapley Additive Explanations (SHAP) framework to quantify the contribution of individual features to the prediction outcomes. Among the five ML algorithm models, the support vectors machine model demonstrated superior discriminative capability, higher goodness-of-fit, and greater clinical utility compared to other ML algorithm models. Moreover, age, systolic blood pressure, total cholesterol, sex, and fasting plasma glucose were the most important risk factors associated with carotid plaques in the MASLD population. This study demonstrated the feasibility of constructing a predictive model for carotid plaques in MASLD populations using health check-up indicators combined with ML algorithms. The application of SHAP methods enhanced model interpretability by quantifying the contribution of individual risk factors to prediction outcomes, enabling clinicians to identify high risk MASLD patients prone to carotid plaque development, so that they can adjust interventions accordingly.

## Introduction

Cardiovascular diseases (CVDs), principally ischemic heart disease and stroke, are the leading causes of global mortality and are major contributors to disabilities^1^. Carotid plaques serve as both a critical subclinical marker of atherosclerosis and a predictor of adverse cardiovascular events^2,3^. Metabolic dysfunction-associated steatotic liver disease (MASLD), affecting 25–30% of adults globally, is increasingly recognized as an independent risk factor for CVDs, with studies reporting a 40–60% prevalence of carotid plaques in MASLD populations^4^. Despite this strong association, current cardiovascular risk stratification tools, such as the Framingham Risk Score, fail to incorporate MASLD -specific biomarkers or leverage advanced predictive analytics, leading to suboptimal risk discrimination in this high-risk cohort^5^.

Recent advances in machine learning (ML) algorithms have demonstrated promising results in clinical prediction models but have been hindered by the “black box” nature of algorithms, limiting their clinical interpretabilities and actionable insights for personalized interventions^6^. The Shapley Additive Explanation (SHAP) is a model-agnostic interpretability method based on the Shapley value concept, derived from cooperative game theory, designed to quantify the contribution of parameters for ML prediction, and to enhance model transparency. By decomposing prediction results into feature contributions, the SHAP transforms complex “black-box” models into explainable frameworks, providing both global and local interpretabilities^7,8^.

However, existing MASLD -focused studies predominantly rely on traditional regression models, which inadequately capture nonlinear interactions among metabolic, inflammatory, and hemodynamic risk factors^9^. Furthermore, while SHAP has been validated in other medical applications to improve model transparency, its application in MASLD -related cardiovascular risk prediction remains unexplored. In this study, we developed and validated a prediction model for the occurrence of carotid plaques in the MASLD population using ML algorithms based on health check-up indicators. The SHAP values were subsequently used to interpret the model’s predictions, revealing the marginal contributions of individual features and their interactions to the risks of carotid plaque development.

## Methods

### Study participants

**Fig. 1:**
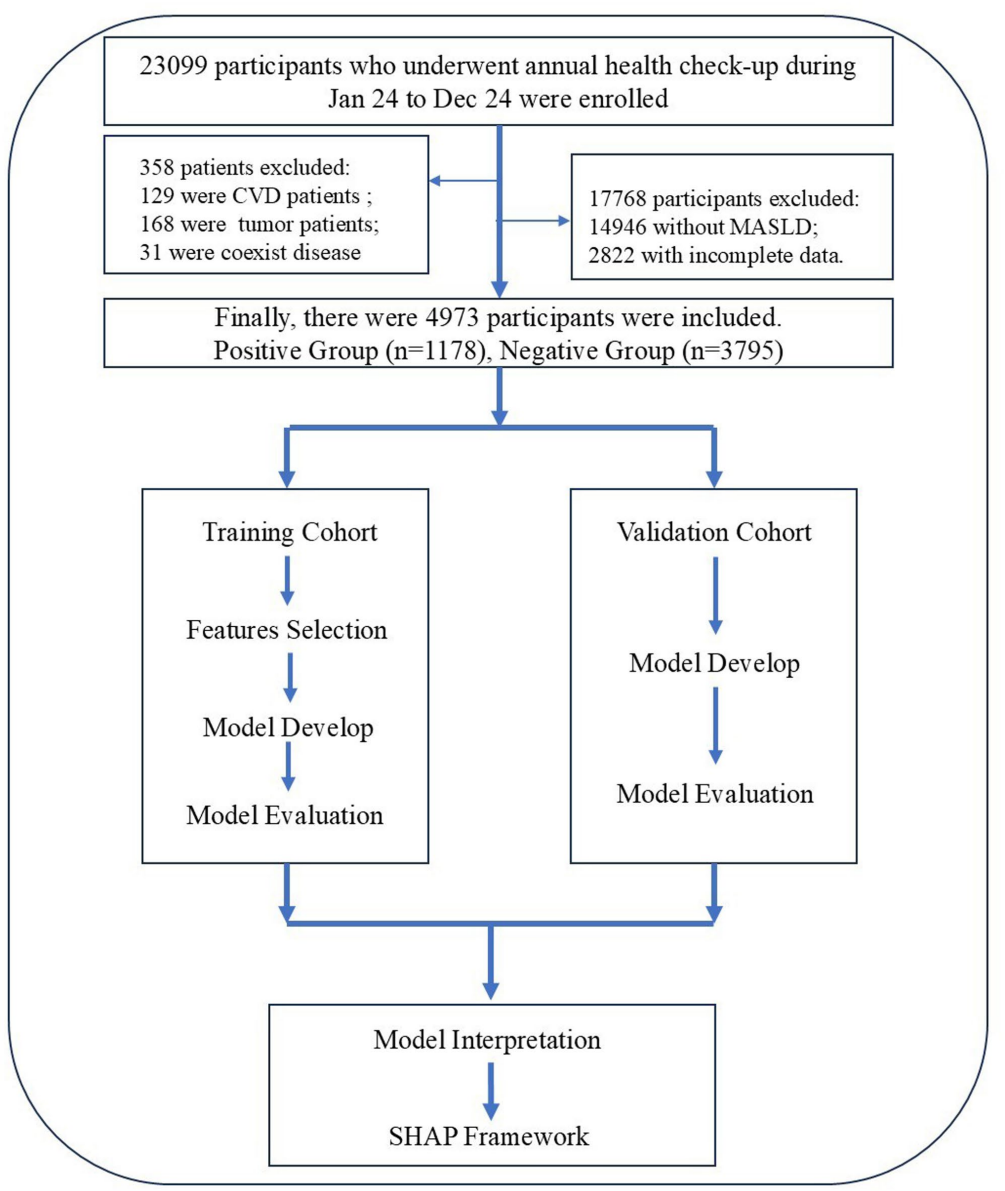
Flow diagram of participant selection, model development, validation, and interpretation.

Participants were enrolled from the annual health check-up population at the Second Hospital of Hebei Medical University, between January 2024 and December 2024. The inclusion criteria were: (1) participants aged ≥ 18 years, and (2) participants with liver ultrasound and carotid ultrasound results with clear diagnostic outcomes. Exclusion criteria included (1) age < 18 years; (2) history of cardiovascular and cerebrovascular diseases or malignant tumors; (3) participants with coexisting etiologies for chronic liver diseases, including hemochromatosis, autoimmune liver disease, chronic viral hepatitis, alpha-1 antitrypsin deficiency, Wilson’s disease, and drug-induced liver injury^10^; (4) Participants missing essential clinical examination indicators (blood tests, biochemistry, anthropometrics); and (5) participants without MASLD. Finally, 4,973 participants were enrolled in this study (Fig. 1).

This study was approved by the Institutional Review Board (IRB) of the Second Hospital of Hebei Medical University (Approval No. 2022-R341). Informed consent was waived by the Institutional Review Board (IRB) of the Second Hospital of Hebei Medical University owing to the study’s retrospective nature. Identifying information was anonymized, adhering to the ethical principles of the Declaration of Helsinki.

### Potential risk features and outcomes

All potential risk factors for carotid plaque reported in recent literature were systematically evaluated. Based on data availability in the study cohort, 26 variables were selected and categorized as follows: demographic characteristics (sex and age), anthropometric measurements [height, weight, body mass index, systolic blood pressure (SBP), diastolic blood pressure (DBP), and pulse]. Blood biochemical indicators were total cholesterol (TC), triglycerides (TG), high-density lipoprotein cholesterol (HDL-C), and low-density lipoprotein cholesterol (LDL-C)]. Metabolic markers were fasting blood glucose (FBG) and uric acid. Hepatic function indicators were alanine transaminase (ALT), aspartate aminotransferase (AST), total protein (TP), albumin (ALB), globulin (GLB), A/G ratio, total bilirubin (TBIL), direct bilirubin (DBIL), and indirect bilirubin (IBIL)]. Renal functions included serum creatinine. Comorbid conditions were hypertension, diabetes mellitus (DM), and hyperlipidemia.

The outcome was whether the participant was diagnosed with MASLD and carotid plaques. The ultrasonographic manifestations of MASLD were primarily characterized by the basic symptoms of steatosis, which increased echogenicity of the liver parenchyma in comparison to the cortex of the right kidney, because intracellular accumulation of fat vacuoles reflected the ultrasound beam^11^. Furthermore, diagnosis of MASLD also met the requirements of the multi-society Delphi consensus statement on new fatty liver disease nomenclature^12^. Carotid artery plaque diagnosis by ultrasound was based on guidelines and standards from the 2020 American Society of Echocardiography^13^.

### Data processing and feature selection

The dataset was randomly divided into a training cohort (*n* = 3,480) and validation cohort (*n* = 1,493), using a 7:3 ratio for model training and internal validation, respectively. All continuous variables underwent Z-score normalization to standardize feature scales, ensuring comparability across parameters. Variables exhibiting significant skewness (Shapiro-Wilk *P* < 0.05) were retained without transformation, leveraging the inherent robustness of tree-based methods to non-normality. The feature selection process and model development were conducted using the training cohort, while the independent validation cohort was used for evaluating the model’s performance.

Correlation between variables was evaluated using Pearson’s (parametric) and Spearman’s (nonparametric) tests. Variables exhibiting absolute correlation coefficients ≥ 0.8 were excluded to mitigate multicollinearity risks. To identify the most predictive features and improve analytical robustness, the least absolute shrinkage and selection operator (LASSO) regression was used for feature selection. This regularization technique applies L1 penalty to shrink less important coefficients toward zero, effectively selecting key variables while mitigating overfitting. The selected features preserved clinically relevant signal strengths, thereby enhancing comparability of latent patterns across datasets.

### Model construction and evaluation

We used three ML algorithms [support vectors machine (SVM), Logistic Regression (LR), Decision Tree (DT), Random Forest (RF) and eXtreme Gradient Boost (XGBoost)] to construct the model based on the features selected by LASSO regression. Hyperparameter optimization was performed via 5-fold cross-validated grid search to ensure robust model performance and reproducibility. For the five ML algorithms evaluated: Support Vector Machine (SVM): Optimized regularization parameter (C: [0.1, 1, 10]) and kernel coefficient (gamma: [0.01, 0.1, 1]); Logistic Regression (LR): Tuned penalty strength (L2 regularization, C: [0.01, 0.1, 1]); Decision Tree (DT): Evaluated maximum depth^3,5,10^ and minimum samples per leaf^5,10^; Random Forest (RF): Optimized number of trees ([100, 200, 300, 400, 500]), features per split ([sqrt(p), log2(p)] where p = number of features), and node splitting criteria ([Gini impurity, entropy]); XGBoost: Calibrated learning rate (η: [0.01, 0.05, 0.1, 0.2]), maximum depth^2–6^, subsample ratio ([0.6, 0.7, 0.8, 0.9, 1.0]), column sampling ([0.6, 0.7, 0.8, 0.9, 1.0]), and minimum loss reduction ([0, 1, 3]).Final configurations were selected by maximizing the AUC on the training cohort through systematic evaluation of all hyperparameter combinations. The optimal parameters were: SVM: C = 10, gamma = 0.1;LR: C = 0.1;DT: max_depth = 5, min_samples_leaf = 10;RF: ntree = 260, mtry = sqrt(p); XGBoost: eta = 0.1, max_depth = 3. The area under the curve (AUC) of receiver operating characteristics (ROC), sensitivity, specificity, accuracy, and F1 score were used to provide a comprehensive assessment of model predictive performance. Furthermore, we used the calibration curve and Decision Curve Analysis (DCA) to evaluate the model’s performance. Calibration curves served as an important tool for ​assessing predictive performance, by juxtaposing predicted probabilities against actual outcomes, thereby validating the model’s alignment with real-world risk profiles. DCA amalgamated ​predictive efficacy and ​clinical utility were used to evaluate a model’s practical value across diverse decision thresholds. By quantifying the ​net benefit (the trade-off between true positives and false positives), DCA identified thresholds where a model’s discriminative power and clinical relevance outperformed simpler heuristics (e.g., “universal treatment” or “no treatment”)^14,15^. Confidence intervals (CI) for performance metrics were derived via 1,000 bootstrap iterations using percentile methods. Stratified sampling maintained original class distributions during resampling.

### Model interpretation

The interpretation of predictive outcomes and the elucidation of feature importance remained critical challenges in machine learning, particularly for complex models where decision-making processes were often opaque. To address these challenges, we employed the ​SHAP framework, a theoretically grounded post-hoc explanation technique rooted in cooperative game theory, to quantify the contribution of each input feature to model predictions. This choice leveraged Shapley values to ensure fairness and consistency in feature attributions, addressing limitations associated with traditional feature importance metrics.

### Statistical analysis

Statistical analysis was conducted using R software version 3.4.3 (http://www.r-project.org). Continuous variables were tested for normality using the Shapiro-Wilk test. If the data conformed to a normal distribution, means ± standard deviations (x ®±s) were reported, and group comparisons were performed using independent sample *t*-tests. If the data were non-normally distributed, medians and interquartile ranges (M, IQR) were presented, with group comparisons conducted using the Mann-Whitney U test. Categorical variables are expressed as counts and percentages (n, %), and group differences were analyzed using the chi square test or Fisher’s exact test.

## Results

### Baseline characteristics

There were 4,973 participants enrolled in this study, with 3,462 males (69.62%) and 1,511 females (30.38%). Among the participants, 1,178 (23.69%) were diagnosed with carotid plaques. Compared to the negative group, the positive group had higher age, SBP, DBP, LDL-C, TC, FPG, urea, UA, TP, ALB, A/G ratio, and AST levels. In addition, the positive group had higher levels of hypertension, DM, and hyperlipemia; but a lower proportion of males (all, *p ≤* 0.05) (Table 1).

**Table 1:** Characteristics of the study population.

Characteristics	N Group(*n* = 3795)	P Group(*n* = 1178)	χ^2^/Z	*p*
Gender			5.079	0.024^a^
Male	2673 (70.43)	789 (66.98)		
Female	1122 (29.57)	389 (33.02)		
Age	41.00,17.00	53.00,13.00	− 27.764	<0.001^b^
BMI kg/m^2^	27.67,4.43	27.68,4.32	− 0.305	0.760^b^
Pulse	81.00,15.00	78.00,15.00	− 7.233	<0.001^b^
SBP (mmHg)	131.00,21.00	140.00,24.00	− 14.003	<0.001^b^
DBP (mmHg)	83.00,16.00	86.00,16.25	− 7.846	<0.001^b^
Hypertension			224.000	0.000^a^
Yes	523 (13.78)	390 (33.11)		
No	3272 (86.22)	788 (66.89)		
DM			79.343	0.000^a^
Yes	178 (4.69)	141 (11.97)		
No	3617 (95.31)	1037 (88.03)		
Hyperlipemia			5.857	0.016^a^
Yes	22 (0.58)	15 (1.27)		
No	3773 (99.42)	1163 (98.73)		
TG (mmol/L)	1.80,1.23	1.81,1.18	− 0.036	0.971^b^
LDL-C (mmol/L)	3.09,1.09	3.17,1.21	− 2.686	0.007^b^
TC (mmol/L)	5.00,1.23	5.14,1.36	− 3.057	0.002^b^
HDL-C (mmol/L)	1.26,0.34	1.29,0.35	− 3.918	<0.001^b^
FPG (mmol/L)	5.24,0.88	5.58,1.51	− 11.763	<0.001^b^
Urea (mmol/L)	4.50,1.36	4.69,1.47	− 6.187	<0.001^b^
Scr (µ mol/L)	78.60,19.90	76.90,19.53	− 1.386	0.166^b^
UA (µ mol/L)	394.00,128.00	370.00,122.00	− 7.248	<0.001^b^
TBIL (µ mol/L)	10.62,6.31	10.92,5.90	− 1.582	0.114^b^
DBIL (µ mol/L)	4.20,1.54	4.28,1.56	− 1.727	0.084^b^
IBIL (µ mol/L)	6.40,4.65	6.53,4.47	− 1.398	0.162^b^
TP (g/L)	73.50,5.20	72.70,5.10	− 6.265	<0.001^b^
ALB (g/L)	46.60,3.50	45.60,3.30	− 11.184	<0.001^b^
GLB (g/L)	26.80,4.50	27.10,4.72	− 1.549	0.121^b^
A/G Ratio	1.74,0.34	1.70,0.33	− 5.617	<0.001^b^
ALT (U/L)	29.30,24.90	25.10,17.67	− 6.471	<0.001^b^
AST (U/L)	24.10,10.90	23.60,10.00	− 1.058	0.290^b^

BMI: body mass index; SBP: systolic blood pressure; DBP: diastolic blood pressure; FPG: fasting plasma glucose; DM: diabetes mellitus; TG: triglycerides; LDL: low-density lipoprotein; TC: total cholesterol; HDL: high-density lipoprotein; UA: uric acid; Cr: creatinine; TBIL: total bilirubin, DBIL: direct bilirubin; IBIL: indirect bilirubin; TP: total protein; ALB: albumin; GLB: globulin; A/G Ratio: albumin-to-globulin ratio.

^a^Chi square (χ^2^) test; ^b^Mann-Whitney U test.

### Comparison of model performance

**Fig. 2:**
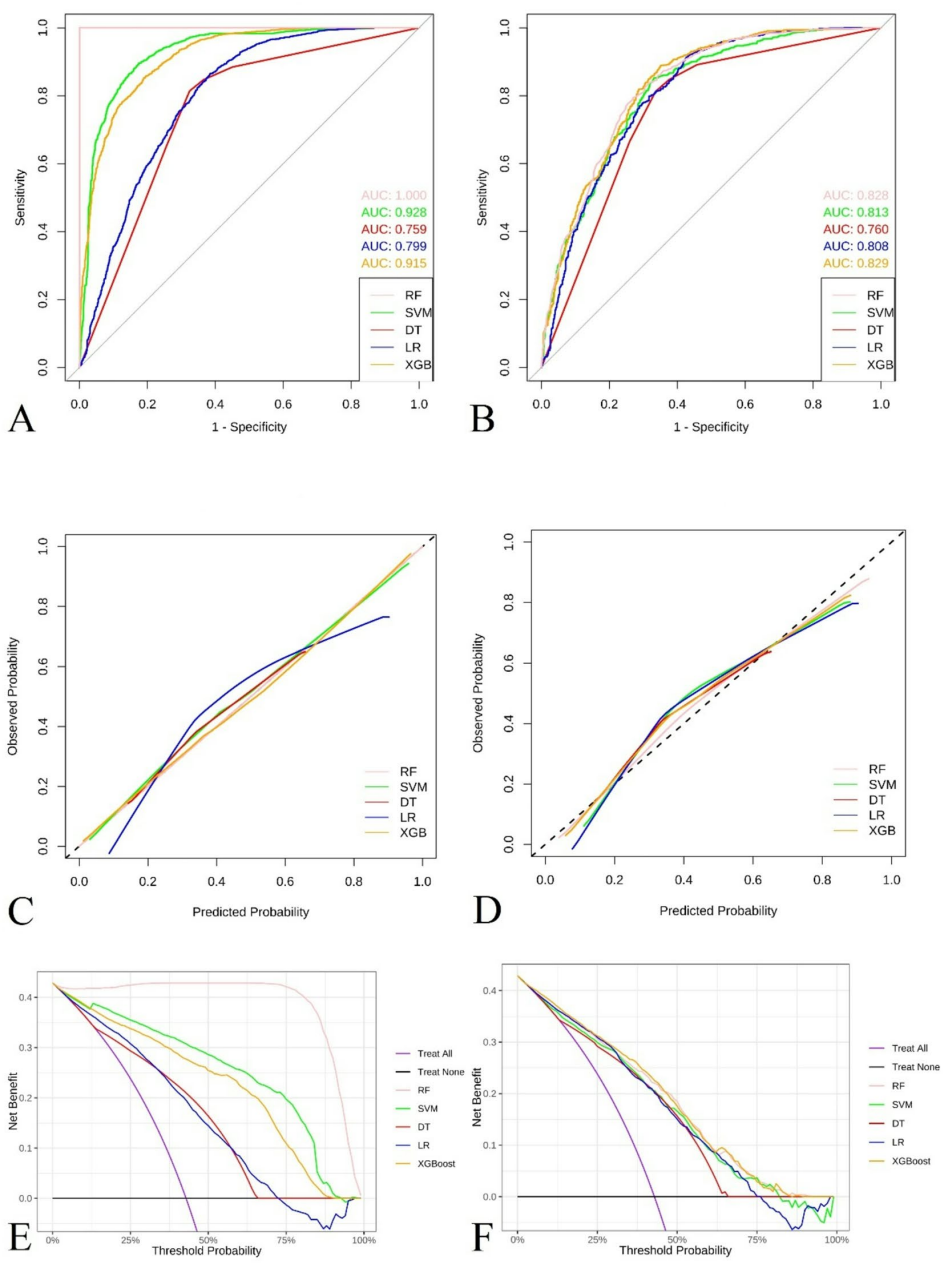
Performance of the predictive model based on three machine learning (ML) algorithm models of the training and test cohorts. (**A**) Receiver Operating Characteristic (ROC) curve of three ML algorithm models of the training cohort. (**B**) ROC curve of three ML algorithm models of the test cohort. (**C**) Calibration curve of three ML algorithm models of the training cohort. (**D**) Calibration curves of three ML algorithm models of the test cohort. (**E**) Decision Curve Analysis (DCA) curve of three ML algorithm models of the training cohort. (**F**) DCA curve of three ML algorithm models of the test cohort.

Table 2 compares five ML algorithms (SVM, DT, LR, RF and XGBoost) across training and validation cohorts. Evaluation metrics included the AUC, sensitivity, specificity, accuracy and F1 score. Among the models, ​SVM demonstrated superior discriminative capability​ (validation AUC = 0.813), while ​XGBoost achieved marginally higher AUC (0.829) but lower specificity (0.744 vs. 0.785)​ (Fig. 2A and B). SVM maintained robust performance across sensitivity (0.674), specificity (0.785), accuracy (0.737), and F1 score (0.773), exhibiting the most balanced clinical utility profile. Calibration curves confirmed SVM’s exceptional reliability, with training and validation predictions closely aligning with observed outcomes (Fig. 2C and D). This concordance with the diagonal reference line indicates precise probability calibration. ​Decision curve analysis further established SVM’s clinical superiority, demonstrating the highest net benefit across decision thresholds (Fig. 2E and F). Notably, RF achieved perfect training metrics (AUC = 1, sensitivity = 1, specificity = 1) but showed reduced validation performance (AUC = 0.828)​, indicating potential overfitting. Despite XGBoost’s strong AUC (0.829), its lower specificity reduced clinical utility for preventive applications requiring minimal false positives. Consequently, SVM was selected as the optimal model​ for carotid plaque prediction in MASLD populations, balancing discrimination (AUC), calibration reliability, and clinical applicability.

**Fig. 3:**
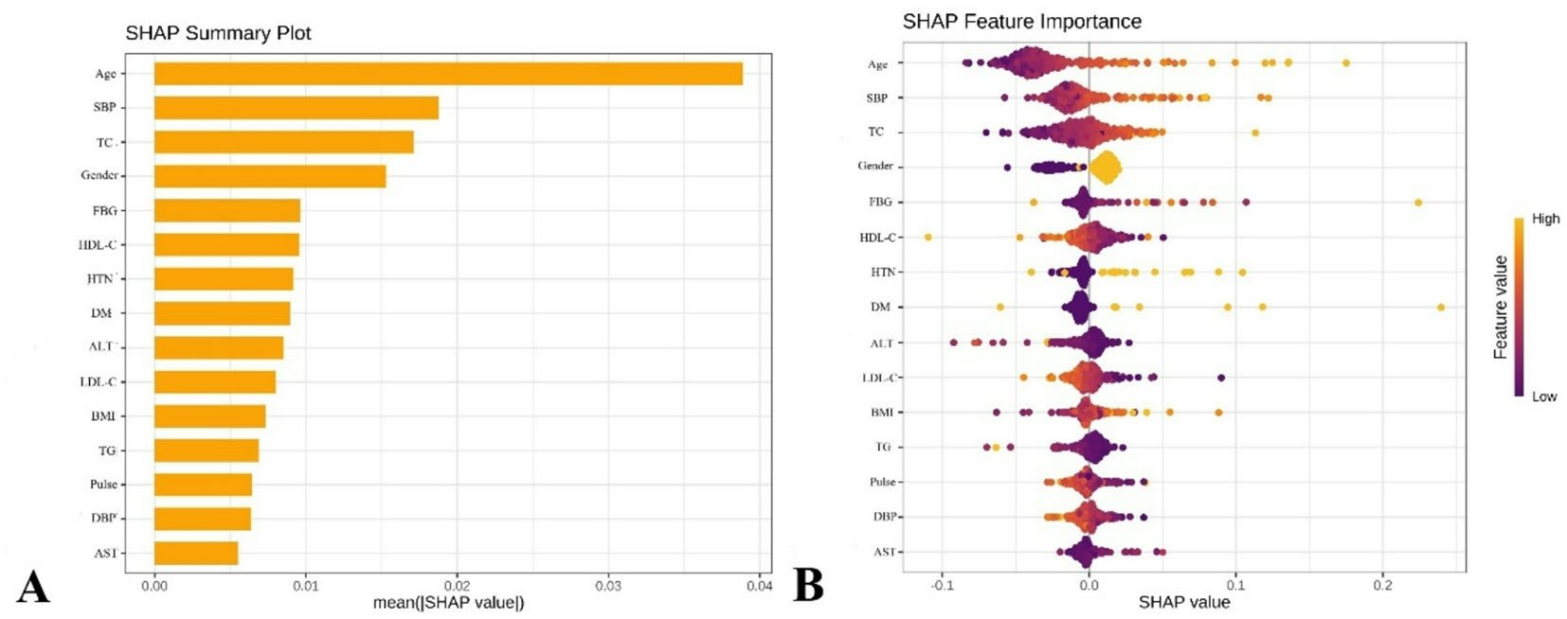
Shapley Additive Explanations (SHAP) interpretation of the support vectors machine model. (**A**) SHAP summary plot. The importance rankings of the model prediction features, and the horizontal coordinates represent the mean absolute SHAP values computed across all samples, where a larger absolute SHAP value indicates a stronger influence of the corresponding features of the model’s predictions. (**B**) SHAP features importance. Each point represents a feature value, and different colors represent the final influence of the feature on the model output results, where yellow represents a larger value and purple represents a smaller value.

Based on the SVM model performance, feature importance was quantified using the absolute SHAP values. The negative and positive contributions of each feature are represented by purple and yellow markers, respectively. The horizontal position of each data point reflects its SHAP value, where higher values indicate stronger contributions to increased predicted probabilities of carotid plaque occurrences, while lower values correspond to reduced risk predictions. Figure 3A and B shows the top 15 features ranked by contribution weights, which are displayed to simplify the interpretation of complex model outputs. This SHAP-based visualization framework enhanced clinical translatability by explicitly mapping the quantitative relationships between key predictors (e.g., age and SBP) and atherosclerotic risk stratification.

**Table 2:** Comparison of the performance of four machine learning methods.

ML	AUC (95%CI)		Sensitivity		Specificity		Accuracy		F1 score	
	Training	Test	Training	Test	Training	Test	Training	Test	Training	Test
SVM	0.928 (0.927–0.935)	0.813 (0.796–0.830)	0.845	0.674	0.867	0.785	0.857	0.737	0.874	0.773
LR	0.799 (0.788–0.810)	0.808 (0.791–0.825)	0.642	0.655	0.771	0.772	0.716	0.722	0.756	0.760
DT	0.759 (0.747–0.771)	0.760 (0.742–0.778)	0.814	0.813	0.676	0.664	0.735	0.728	0.745	0.736
RF	1	0.828 (0.812–0.844)	1	0.730	1	0.770	1	0.753	1	0.781
XGBoost	0.915 (0.909–0.922)	0.829 (0.813–0.845)	0.838	0.751	0.816	0.744	0.826	0.747	0.843	0.771

SVM: Support Vector Machine; LR: logistic regression; DT: Decision Tree; RF: Radom Forest.

## Discussion

In recent years, numerous studies have reported a significant association between MASLD and carotid plaque formation^16–18^. This growing body of evidence underscores the critical role of MASLD as a predictor of subclinical atherosclerosis and cardiovascular risk, particularly through mechanisms involving metabolic dysregulation and systemic inflammation^16^. Notably, longitudinal cohort studies have reported that both the presence and progression of MASLD, especially in advanced fibrosis stages, are independently associated with increased carotid plaque burden^19^. These findings highlight the need for integrated cardiovascular risk assessment in MASLD patients, to mitigate the burden of ischemic cerebrovascular events.

ML algorithms have revolutionized clinical prediction model development by facilitating the integration of complex, high dimensional data, to improve diagnostic accuracy, risk stratification, and therapeutic decision-making^20,21^. However, the development of predictive models for carotid plaques in the MASLD population, which integrates health check-up indicators with ML algorithms, remains poorly understood. Deng Y, et al.^9^ developed a prediction model for carotid plaques based on the health check-up indicators of 5.4 million adults with fatty liver disease, combined with ML algorithms. The predictive model showed good performance in an internal validation set (AUC = 0.831) and external validation set (AUC = 0.801). The model also graphically showed good calibration capabilities. However, this predictive model has not undergone validation using ​DCA to assess its clinical utility, nor has it undergone SHAP analysis for interpretability characterization. In addition, the study population comprised individuals with ​fatty liver disease, rather than MASLD.

In the present study, we constructed a predictive model for carotid plaques in the ​ MASLD population, based on ​health check-up indicators, which showed ​efficiency, ​straightforwardness, and ​practicality in clinical applications. Among the three ML algorithms, SVM demonstrated the best performance, achieving an AUC of 0.813 in the validation cohort, The model exhibited favorable calibration characteristics, goodness-of-fit properties, and clinical usefulness as shown by the calibration and DCA curves, which exhibited ​efficiency, ​straightforwardness, and ​practicality in clinical applications. Notably, while SVM demonstrated strong discriminative capability (AUC = 0.813), XGBoost achieved marginally superior performance in the validation cohort (AUC = 0.829). This aligns with prior studies where boosting algorithms outperformed kernel-based methods in complex biomedical prediction tasks^22^. However, SVM maintained higher specificity (0.785 vs. 0.744), suggesting greater reliability in identifying low-risk individuals. Given the clinical priority of minimizing false positives in preventive cardiology, SVM’s balanced performance profile – coupled with its superior interpretability via SHAP – justified its selection as the primary predictive tool. To resolve persistent challenges in ML algorithm interpretability, and to enable transparent visualization of prediction determinants, we implemented SHAP in our SVM model, to systematically assess both ​global (feature importance rankings) and ​local (individual prediction analyses) interpretabilities. This integration enabled us to identify critical features driving predictions, such as age, SBP, TC, sex, and FBG, which were important features in prediction of carotid plaques in the MASLD population, while also revealing nonlinear relationships and interaction effects that may be masked by simpler methods. The computed SHAP values quantified directional feature contributions, with positive scores indicating elevated carotid plaque risks associated with specific feature values in the MASLD population, while negative values indicated protective effects. Through tree-specific implementation of the SHAP’s additive feature attribution framework, our methodology enabled granular visualization of nonlinear decision pathways, bridging the gap between algorithmic complexity and clinical reasoning.

The results in the present study, which showed that age, SBP, TC, sex, and FBG were the most important features of carotid plaques in MASLD populations, were consistent with previous studies^9,23^. Aging induces cumulative metabolic and vascular stress, fostering carotid plaque formation through three principal mechanisms: (1) age-related reactive oxygen species accumulation and elevated pro-inflammatory cytokines (TNF-α and IL-6), which disrupt endothelial integrity, promote lipid oxidation, and drive foam cell formation, initiating atherosclerotic plaque development^24^; (2) declining nitric oxide bioavailability and arterial stiffening impair vasodilation, enhance platelet aggregation, and potentiate thrombotic events^25^; and (3) reduced insulin sensitivity exacerbates hepatic steatosis and systemic metabolic imbalance, accelerating atherosclerosis progression^26^. Hypertension is a well-established risk factor for carotid atherosclerosis^27,28^. Elevated SBP accelerates vascular endothelial injury and promotes LDL-C infiltration into the arterial wall, leading to plaque initiation and progression^27^. In addition, elevated SBP results in arterial stiffness and promotes cytokine release (IL-6, TNF-α), enhancing monocyte adhesion and intraplaque inflammation^29^. Furthermore, high cholesterol is a major risk factor for carotid artery disease, which can lead to narrowing or blockage of the carotid arteries supplying blood to the brain; and which also plays a role in the progression of carotid artery stenosis and overall cardiovascular risk^30^. The mechanism involves: (1) small, dense LDL particles infiltrating arterial intima, undergoing oxidation, and being phagocytosed by macrophages to form foam cells, which are the core of atherosclerotic plaques^31^; and (2) reduced HDL-C, impairing reverse cholesterol transport, and failing to clear lipid-laden macrophages from plaque sites^32^. In addition, males with higher occurrences of carotid plaques are characterized by the following: (1) higher testosterone levels in males promote insulin resistance and accelerate atherosclerosis, but females with higher estrogen can enhance or reverse cholesterol transport and reduce vascular inflammation^33^. (2) In male MASLD patients, oxidized LDL preferentially infiltrates the arterial wall of males, where it is engulfed by macrophages via scavenger receptors, leading to foam cell formation and the release of pro-inflammatory chemokines (e.g., MCP-1)^34^. This process recruits’ monocytes to the arterial intima, causing plaque initiation and progression, while reduced paraoxonase-1 activity impairs HDL’s antioxidant capacity, diminishing its ability to detoxify oxidized LDL and mitigate endothelial dysfunction. This further exacerbates vascular inflammation and plaque vulnerability^35^. Some studies had reported that elevated FBG increases advanced glycation end-products (AGEs), which bind to the receptor for AGEs on endothelial cells, activating the NF-κB pathway and promoting inflammatory cytokines (e.g., TNF-α and IL-6). This accelerates LDL oxidation and foam cell formation^36^.

### Limitations

There were several limitations in this study. First, this study has several inherent limitations. The single-center retrospective design and cross-sectional nature preclude establishing causal or temporal relationships between MASLD and carotid plaque occurrence. The restricted one-year temporal scope constrains longitudinal assessment. Potential selection bias may affect population representativeness and residual confounding; Model generalizability requires validation in multi-ethnic cohorts and prospective settings; Future research should prioritize multicenter prospective cohorts to validate prediction performance temporally, establish causal mechanisms, and explore advanced architectures (e.g., transformer networks) for feature representation learning. Second, the diagnosis of MASLD was defined by ​ultrasonography rather than liver biopsy. However, studies have reported a strong correlation between ultrasonographic findings and histopathological results from liver biopsies, particularly in detecting hepatic steatosis^37^. Finally, SHAP analysis does not quantify the importance of predictors in real quant problems, but rather their importance to the model’s predictions^38^.

## Conclusions

This study developed and validated predictive models for carotid plaque occurrence in patients with MASLD by integrating demographic data, blood biochemical indices, and clinical parameters from annual health check-up populations, using ML algorithms. The SVM-based models showed high accuracy and robust reliability in predicting carotid plaque development. Furthermore, we used advanced SHAP technology for model interpretation and visualization, to facilitate a precise characterization of risk factors for carotid plaques in MASLD populations. By combining SHAP technology with ML algorithms, we quantified the contributions of key risk factors such as age, SBP, TC, sex, and FBG to carotid plaque risk, thereby possibly providing precise clinical insights into the management of carotid plaques associated with MASLD patients.

## Data Availability

The datasets generated and/or analyzed during this study are available from the corresponding author upon reasonable request.
